# Integrated optical and thermal modeling for the development of a scalable multi-sample light-assisted drying platform for biologic stabilization

**DOI:** 10.3389/fbioe.2026.1844186

**Published:** 2026-06-09

**Authors:** Anteneh A. Tsegaye, Alexander J. Suptela, Russell G. Keanini, Susan R. Trammell

**Affiliations:** 1 Department of Physics and Optical Science, University of North Carolina at Charlotte, Charlotte, NC, United States; 2 Department of Biological Sciences, University of North Carolina at Charlotte, Charlotte, NC, United States; 3 Department of Mechanical Engineering and Engineering Science, University of North Carolina at Charlotte, Charlotte, NC, United States

**Keywords:** biopreservation, light-assisted drying, multiple-sample processing, optical modeling, thermal modeling, thermal stabilization

## Abstract

**Introduction:**

Light-Assisted Drying (LAD) is a new technique for stabilizing biologics such as vaccines and protein-based therapeutics by using near-infrared laser radiation to drive selective water removal and encapsulate materials within a protective sugar matrix. This process enables room-temperature storage of biological products and reduces dependence on costly cold-chain logistics. While previous studies utilized single-sample LAD, this work introduces a multi-sample platform for the first time. By integrating optical and thermal modeling, we demonstrate that 1,064 nm radiation allows for energy reuse across vertically stacked samples to enable scalable, high-throughput processing.

**Methods:**

LightTools optical simulations were used to model laser energy deposition across vertically stacked samples. A lumped-parameter thermal model was developed to predict temperature evolution and drying behavior during laser irradiation. Together, these models guided the design of a processing system. A prototype system was subsequently constructed and tested.

**Results:**

LightTools simulations revealed that each sample absorbed <10% of incident laser power, enabling a vertical stacking configuration. A lumped-parameter thermal model was developed which predicted that a 5 W near-infrared laser could process a three-sample stack within 100 min while maintaining peak temperatures below 33 °C. To validate these models, a prototype system was constructed and tested using Human Immunoglobulin G (Human IgG) as a model biologic. Experimental results demonstrated that all samples dried within 100 min without exceeding the thermal denaturation threshold. Enzyme-Linked Immunosorbent Assay (ELISA) confirmed that processed Human IgG retained full binding affinity ( p>0.05 compared to unprocessed controls), and Differential Scanning Calorimetry (DSC) showed no significant changes in the protein’s melting temperature $(T_{m}\approx$ 73 °C) or unfolding enthalpy (ΔH) .

**Discussion:**

These findings demonstrate the feasibility of a high-throughput LAD system and represent an important step toward its industrial-scale implementation for biologic preservation.

## 1 Introduction

Vaccination against infectious disease is one of the greatest advances of modern medicine as evidenced by the elimination of smallpox worldwide and the prevention of an estimated 2.5 million deaths per year from diphtheria, whooping cough, and measles (Roush et al., 2007; World Health Organization, 2013; Oyston and Robinson, 2012; Enserink, 2010). However, vaccines are temperature-sensitive biologics and the vast majority of them must be stored between 2 and 8 °C. Temperature excursions above or below this recommended temperature range can decrease their potency (Matthi et al., 2007; Levin et al., 2007; Center for Disease Control National Center for Immunization and Respiratory Diseases, 2012). Similarly, protein-based therapeutics, including monoclonal antibodies, have emerged as effective treatments for a range of conditions such as cancers, autoimmune diseases, and infectious diseases (Tambuyzer et al., 2020; Lu et al., 2020; Wang et al., 2022; Delgado and Garcia-Sanz, 2023). However, these therapies also require cold storage to maintain their stability and efficacy. In addition, DNA and RNA are biobanked for research and clinical applications, such as gene expression studies and RNA-based vaccines (Seyhan, 2024; Rao, 2022; Edsjö et al., 2023). These molecules are similarly sensitive to temperature and require temperature-controlled storage conditions to prevent degradation. Cold storage strategies are expensive and especially burdensome in low-resource settings due to a lack of available infrastructure. The global healthcare cold chain logistics market was valued at $62.5 billion in 2025, with projections reaching $95.1 billion by 2030 (Mordor Intelligence, 2025a). Despite this growth, temperature excursions remain a critical failure point, costing the industry approximately $35 billion annually in lost product and waste (Mordor Intelligence, 2025b) Furthermore, refrigerated warehouses are among the most energy- intensive industrial spaces, with refrigeration accounting for up to 70% of total electricity consumption and utilizing four to five times more energy per square foot than standard commercial buildings (Mordor Intelligence, 2025a). Such high operational costs and energy demands highlight the need for ambient-temperature stabilization technologies like LAD.

Lyophilization (freeze-drying) is used to thermally stabilize some biologics such as vaccines and protein-based therapeutics (Hassett et al., 2015; Hassett et al., 2013; Naik et al., 2017; Wang, 2000; Clausi et al., 2008). However, often these types of products are damaged during the required freezing step of processing and cannot be lyophilized. Furthermore, this technique requires long processing times (>24 h) and formulations with multiple components that can increase the cost and complexity of the product. Spray drying and foam drying have also had limited success in stabilizing biologics (Ohtake et al., 2011; Abdul-Fattah et al., 2007; Fourie et al., 2008; Chen et al., 2010; Huyge et al., 2012; Wong et al., 2007). Freeze drying, spray drying, and foam drying all expose biologics to extreme temperatures and/or pressure conditions. There is a need for new techniques to produce thermally stable biologics that can overcome these challenges.

**FIGURE 1:**
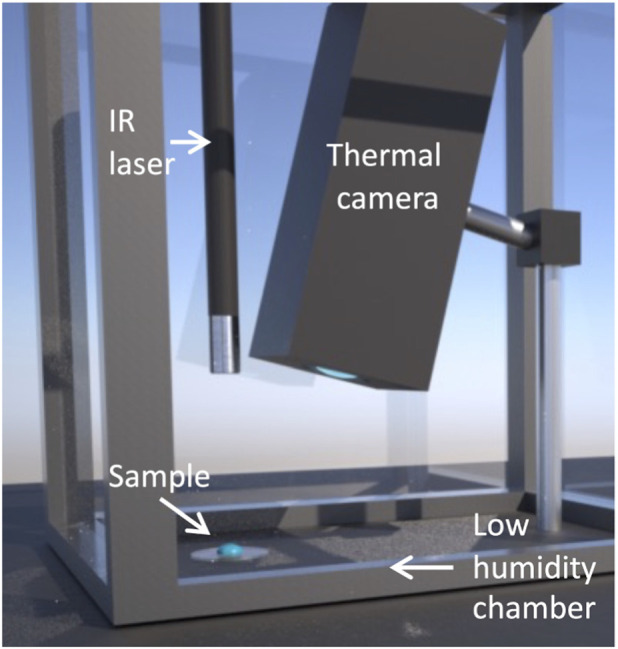
Current experimental set-up for LAD processing samples one at a time. Samples are illuminated with a near-IR laser from above. The thermal camera is used to monitor the sample temperature and the power meter monitors transmitted laser power during processing. All samples are processed inside a low humidity chamber (adapted from (Young et al., 2018)).

We have developed a new drying technique called light-assisted drying (LAD) to thermally stabilize biologics. Water is selectively heated *via* near-infrared laser (1,064 nm) illumination, rapidly removing water from a sample, and forming an amorphous matrix that can be stored at supra-zero temperatures (see Figure 1). During the dehydration process, sugar molecules substitute for water *via* hydrogen bonding and mitigate the risk of protein aggregation and unfolding (Chen et al., 2022; Crowe et al., 1998; Crowe et al., 1984; Carpenter and Crowe, 1989). The resulting vitrified state protects the biomolecule by restricting molecular mobility and arresting the diffusion-controlled processes that lead to degradation. LAD has been successfully applied to the model protein lysozyme and nucleic acid nanoparticles. Samples were LAD processed and then stored at room temperature. The function/structure of the embedded biologic was confirmed after storage (Young et al., 2018; Young et al., 2020; Anh Lam et al., 2022; Furr et al., 2024; Tsegaye et al., 2024). Most recently, LAD was applied to a commercially available poliovirus vaccine, demonstrating its applicability to clinically relevant biologic formulations (Tsegaye et al., 2025). These studies indicate that LAD can effectively stabilize biologics for storage at ambient temperatures.

Currently, samples are LAD processed one at a time. This study investigates strategies to enable the simultaneous processing of multiple samples using LAD. LightTools optical modeling software was employed to simulate light propagation and energy deposition within samples during LAD and to design a system capable of processing multiple samples concurrently. A lumped-parameter thermal model was developed to estimate laser-induced heating and the corresponding evaporative, convective, and conductive cooling effects. This model tracks changes in sample water content as a function of laser parameters, enabling prediction of drying behavior. Together, the optical and thermal simulations establish a framework for designing systems capable of efficient, multi-sample LAD processing. To experimentally validate the models, a multi-sample processing prototype was constructed and tested in the laboratory. Using this setup, LAD was applied to samples containing the protein human immunoglobulin G (IgG). Post-processing, protein functionality was assessed using an enzyme-linked immunosorbent assay (ELISA), while structural integrity was evaluated through differential scanning calorimetry (DSC).

## 2 Materials and methods

### 2.1 LAD processing

The LAD system for single-sample processing is shown in Figure 1. The same laser, humidity chamber and thermal camera were used for the development of the multi-sample system described in Section 3. A continuous-wave ytterbium fiber laser (IPG Photonics, YLR-5-1,064; maximum power output, 5 W) with a Gaussian beam is used to process samples within a low-humidity chamber. The laser’s power output and beam spot size (4.5 mm FWHM) were measured using a thermal sensor (Beam Track 10A-PPS, Ophir Photonics). Temperature monitoring during processing is conducted using a mid-infrared camera (FLIR A6 series). The power meter is used to monitor transmitted laser power during processing. All experiments are performed in a humidity-controlled environment with approximately 2% relative humidity (RH). Samples consist of a biologic suspended in a drying solution containing the matrix forming sugar, trehalose. The laser energy is absorbed by the water in the sample, resulting in accelerated evaporation. As the water is removed, an amorphous trehalose preservation matrix is formed. Samples are processed as droplets (volumes up to 0.25 mL) on glass coverslips or in glass vials (volumes > 0.25 mL) commonly used in industry (see (Young et al., 2018; Young et al., 2020; Anh Lam et al., 2022; Furr et al., 2024; Tsegaye et al., 2024)) The limitation of this system is that only one sample is processed at a time.

### 2.2 Modeling light propagation through and energy deposition into samples during LAD processing Using LightTools

**FIGURE 2:**
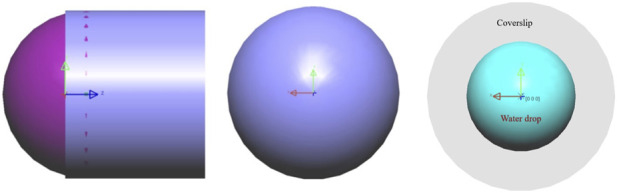
Composite sphere-cylinder assembly pre-subtraction (left panel) and resultant hemispherical water droplet geometry post-subtraction (center panel). Illustration of the final geometry of the droplet (blue) with the coverslip (right panel).

All modeling of the light propagation was done using LightTools (version 2022.03, Synopsys, Inc.). Samples were modeled as droplets on glass coverslips because this geometry has been used in previous experiments. Water is the primary absorber of laser energy during the drying process. To simulate a water droplet in LightTools, solid cylinders and spheres were used. A sphere with a 6 mm radius and a cylinder with a 6 mm radius and 10 mm length were created. The cylinder was placed at the origin (0, 0, 0) of the coordinate system, and the sphere was positioned at (0, 0, 1.5). A 1.5 mm segment was removed from the lower portion of the sphere to yield a hemispherical droplet with a maximum thickness of 4.5 mm and a radius of 6 mm. These are the dimensions for a 0.25 mL droplet geometry used in prior LAD experiments. The droplet was assigned the optical properties of water at 1,064 nm: a refractive index of 1.326 and an absorption coefficient of 0.015 ${\text{mm}}^{-1}$ (Kedenburg et al., 2012). Fresnel losses were also included. A borosilicate glass coverslip was modeled as a 2.2 cm diameter, 1 mm thick disk matching the coverslips used in LAD experimental studies. The coverslip was assigned a refractive index of 1.51 and an absorption coefficient of $8.9\times 10^{-5}$ ${\text{mm}}^{-1}$ at 1,064 nm, consistent with the properties of borosilicate glass (Schott, 2024). A Boolean operation was then used to merge the droplet and coverslip models, ensuring direct contact between the surfaces. The final LAD droplet geometry is shown in Figure 2.

**FIGURE 3:**
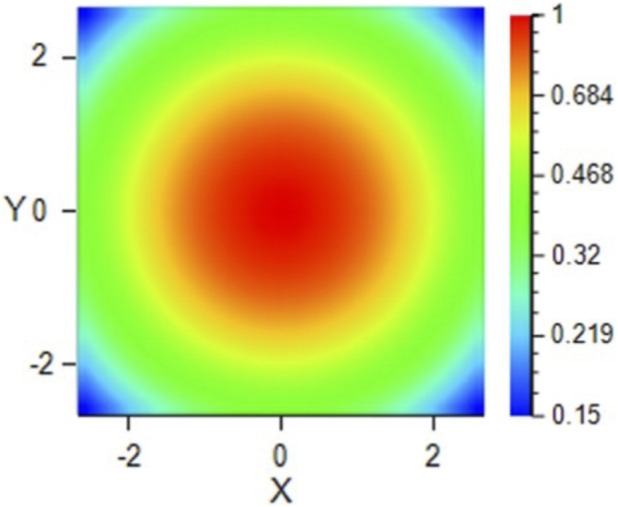
Light source normalized intensity on a log scale. The source is a rotationally symmetric Gaussian laser beam with a full width at half maximum (FWHM) of 4.5 mm. The X and Y-axes represent physical coordinates in millimeters, and the color scale denotes normalized intensity, with the peak value set to 1.

The light source used in the simulations was configured to match the properties of the 1,064 nm laser employed in experimental studies. It was assigned a radiometric power output of 5 W and modeled as a collimated beam with a Gaussian spatial distribution, characterized by a full width at half maximum (FWHM) of 4.5 mm. The beam exhibited uniform angular distribution, rotational symmetry, and an outward trace direction. The resulting beam spot had a surface area of 25.518 ${\text{mm}}^{2}$ . The profile of the simulated light source is shown in Figure 3.

The modeled sample geometry was combined with the defined light source for the simulations of LAD. For all models, a Monte Carlo simulation was performed to evaluate light propagation through the sample. Additional optical elements, such as lenses and mirrors, can be incorporated as needed to steer, focus, or collimate the laser beam for optimized energy delivery.

### 2.3 Lumped-parameter thermal modeling of a laser-heated droplet on a coverslip

To estimate the maximum sample temperature reached during processing and the time required for complete water removal, both as a function of incident laser power, a lumped-parameter thermal model was developed. The model was used to predict the time-dependent change in sample volume and estimate total drying duration needed to achieve low (1%–2%) water content by mass. During LAD, a water-based liquid sample is irradiated with a near-infrared laser and the absorbed energy raises the temperature of the water to initiate evaporation. Water was used in the thermal model because it is the primary target for removal in order to create the preservation matrix. Its well-characterized thermal properties facilitated a detailed analysis of key heat transfer mechanisms, including laser absorption, conduction, convection, and evaporative cooling. In previous experimental studies, samples have been LAD processed as droplets (0.25 mL) on glass coverslips, so this geometry was simulated. The lumped-parameter approach is justified by experimental IR thermal imaging of the 0.25 mL droplets, which showed no significant temperature gradients across the sample during laser irradiation. These observations confirm that internal heat conduction is sufficiently rapid to maintain thermal uniformity, allowing the droplet to be accurately modeled as a single thermal mass. The model provided time-resolved simulations of evaporation-driven drying and predictions of drying time under varying laser power conditions. The model focuses on the active evaporation phase, as the process is terminated once bulk evaporation has ceased. Equation 1 describes the time-dependent temperature change of the water forming the droplet, with all variables defined in Table 1.

$$\frac{dT_{w}}{dt}=\frac{a_{w}S_{w}-h_{T}A_{T}\left(T_{w}-T_{\infty }\right)-h_{B}A_{B}\left(T_{w}-T_{s}\right)-\varepsilon_{w}\sigma A_{T}\left(T_{w}^{4}-T_{\infty }^{4}\right)-\dot{m_{w}}h_{fg}}{\rho_{w}c_{pw}V_{w}}$$ (1)

**TABLE 1:** List of symbols and descriptions in the governing equation of the lumped-parameter thermal model.

Symbol	Description	Units
$\rho_{w}$	Density of water	kg/m^3^
$c_{pw}$	Specific heat capacity of water	J/(kg ⋅ K)
$T_{w}$	Time-dependent temperature of the water droplet	° C
$T_{s}$	Time-dependent temperature of the glass substrate	° C
$T_{\infty }$	Ambient temperature	° C
$V_{w}$	Time-dependent water droplet volume	${\text{m}}^{3}$
$S_{w}$	Laser power incident on the water droplet	W
$a_{w}$	Absorption efficiency of water	-
$A_{T}$	Top surface area of the water droplet	${\text{m}}^{2}$
$A_{B}$	Contact area between water and glass slide	${\text{m}}^{2}$
$h_{T}$	Convective heat transfer coefficient (water-air interface)	W/( ${\text{m}}^{2}\cdot$ K)
$h_{B}$	Interfacial heat transfer coefficient (water-substrate)	W/( ${\text{m}}^{2}\cdot$ K)
$\varepsilon_{w}$	Thermal emissivity of water	-
σ	Stefan-Boltzmann constant	W/( ${\text{m}}^{2}\cdot {\text{K}}^{4}$ )
${\dot{m}}_{w}$	Mass loss rate of water due to evaporation	kg/s
$h_{fg}$	Latent heat of vaporization of water	J/kg

Five terms govern the behavior of the droplet. The first term, $a_{w}S_{w}$ , represents heating due to the absorption of incident laser energy by the droplet. The second term, $h_{T}A_{T}\left(T_{w}-T_{\infty }\right)$ , accounts for convective heat loss from the droplet’s top surface to the surrounding air. The third term, $h_{B}A_{B}\left(T_{w}-T_{s}\right)$ , represents heat transfer between the bottom of the droplet and the glass coverslip. The fourth term, $\varepsilon_{w}\sigma A_{T}\left(T_{w}^{4}-T_{\infty }^{4}\right)$ , describes radiative cooling of the droplet. The final term, ${\dot{m}}_{w}h_{fg}$ , quantifies evaporative cooling losses as water escapes from the droplet.

An important consideration for our model is that the relative humidity within the LAD processing chamber is low (RH ≈ 2%). The large vapor pressure gradient between the saturated water vapor at the droplet surface and the dry ambient air drives rapid evaporation (Incropera et al., 2007). As water molecules escape from the droplet surface, they carry away latent heat, leading to a reduction in the droplet’s temperature. Conduction and convection play comparatively minor roles in this environment due to the limited thermal mass and the rapid rate of phase change. Consequently, evaporative cooling governs the thermal behavior of the droplet during the LAD process and the governing equation can be simplified to

$$\frac{dT_{w}}{dt}=\frac{a_{w}S_{w}-\dot{m_{w}}h_{fg}}{\rho_{w}c_{pw}V_{w}}.$$ (2)

Mass loss caused by evaporation can be modeled by Equation 3 that links the rate of volume decrease to the evaporation rate.

$$\frac{dm_{w}}{dt}=\rho_{w}\frac{dV_{w}}{dt}=-{\dot{m}}_{w}$$ (3)

Equation 2 can then be written as

$$\frac{dT_{w}}{dt}=\frac{a_{w}S_{w}+\rho_{w}\frac{dV_{w}}{dt}\cdot h_{fg}}{\rho_{w}c_{pw}V_{w}}$$ (4)

The heating and cooling of the glass coverslip were not incorporated into the present model, as their effects become relevant only after the water has fully evaporated. These factors will be addressed in future studies.

### 2.4 LAD processing using IgG as a representative biologic

Immunoglobulin G (IgG) was used as a representative biologic for processing. IgG was suspended in a 0.2 M disaccharide trehalose drying solution (DS) in 0.33 x phosphate buffer solution (PBS) resulting in a protein concentration of 0.50 mg/ml. A droplet (0.25 mL) was then deposited on a glass coverslip (Fisher brand 12–5,462) for LAD processing. Three samples were simultaneously processed using a 5 W 1064 nm laser (IPG Photonics, YLR-5-1,064) and were irradiated for 2 h and 20 min. Five groups of three samples were processed, yielding a total of 15 samples (the design of this multi-sample setup is described in Section 3). The dried samples were then stored at room temperature for 2 months.

Direct Enzyme-Linked Immunosorbent Assays (ELISAs) were performed to evaluate the ability of a commercially available secondary antibody against human IgG (Thermo Fisher, catalog #05–4,220) to detect human IgG subjected to LAD processing. Both unprocessed and LAD-processed human IgG samples, stored for 2 months, were coated onto ELISA plates overnight, followed by incubation with an HRP-conjugated mouse anti-human IgG secondary antibody. IgG samples heat-treated at 98 °C for 2 h were included as a negative control. Absorbance data from the ELISAs were evaluated for normality and homogeneity of variance prior to statistical comparison. The Lilliefors test was used to determine whether each group was normally distributed (Lilliefors, 1967), and Levene’s test was used to assess equality of variances across groups (McDonald, 2014). If the data were normally distributed and variances were equal, a standard one-way ANOVA was applied, followed by Tukey’s HSD *post hoc* test to evaluate pairwise differences between groups (McDonald, 2014). If the data were normally distributed but variances were unequal, Welch’s ANOVA was applied, as it accommodates heteroscedasticity and unequal sample sizes (Welch, 1951), with *post hoc* comparisons performed using the Games–Howell test (Games and Howell, 1976). For all analyses, results were considered statistically significant at p<0.05 .

To evaluate the structural integrity of the IgG protein following LAD processing, differential scanning calorimetry (DSC) was conducted using a microcalorimeter (MicroCal VP-DSC, Northampton, MA). DSC was performed on four groups: unprocessed samples (N = 3), LAD-treated samples analyzed immediately after processing (N = 3), non-crystallized LAD-treated samples stored for 2 months at 20 °C (N = 3) and a LAD processed sample that crystallized during storage (N = 1). All samples were rehydrated in 0.33 x phosphate buffer solution (PBS) prior to DSC measurement. The IgG concentration after rehydration was 0.002±0.001 mM, and DSC scans were normalized accordingly. Baseline stability was verified by performing at least 10 scans of the buffer reference solution. Once a stable baseline was achieved, the IgG-containing samples were loaded into the calorimeter *via* syringe. Scans were performed from 10 °C to 90 °C at a rate of 70 °C/h, following a 15-min pre-scan equilibration. Data analysis was performed using Origin software (MicroCal) to determine the midpoint transition temperature $\left(T_{m}\right)$ and the calorimetric enthalpy of unfolding (ΔH) .

The onset of crystallization serves as an indicator of matrix instability. Therefore, polarized light imaging (PLI) was conducted to detect potential crystal inclusions that may have formed during LAD processing or subsequent storage, as described previously (Tsegaye et al., 2024). The PLI set-up consisted of a white light fiber optic illuminator (41,720, Cole Palmer), two linear polarizers (LPVISE050-A, Thorlabs) with the second polarizer acting as an analyzer, and a digital camera (Nikon D100) aligned in the vertical direction. The spatial resolution of the set-up was 10 μ m/pixel. Samples were placed on a glass slide between the polarizers and imaged from above. Two images were taken: the first with the analyzer oriented at 0 ° to the polarizer and the second with the analyzer oriented at 90 ° with respect to the polarizer. For each sample, images were taken immediately after processing and after storage.

## 3 Results

### 3.1 Absorption of laser energy in samples

**FIGURE 4:**
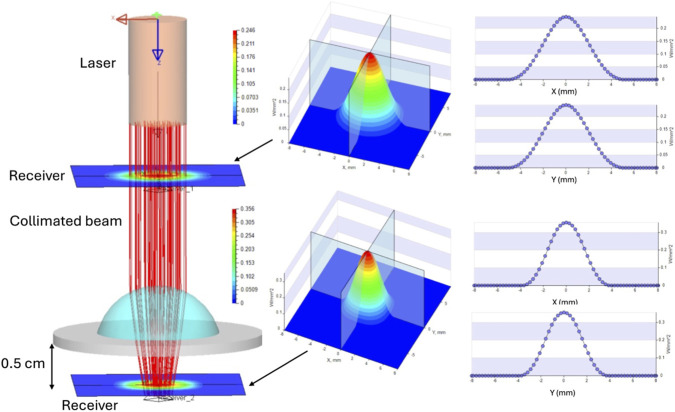
LightTools model of the light source, droplet sample and coverslip. The receivers were integrated into the model to determine the power and beam profile at various positions. The Cartesian isometric views show the light distribution before and after the light travels through the drop.

The droplet + coverslip and the light source were used to simulate the LAD process in LightTools. Figure 4 shows the simultaion for a single sample and consists of the light source and the droplet/coverslip. The blue squares in the figure are receivers used in the simulation to calculate the laser power and laser beam profile at specific positions. The receiver before the sample measured an input power of 5 W and irradiance $0.33\;{\text{W/mm}}^{2}$ . The beam diameter was 7.65 mm (measured at $1/e^{2}$ intensity) and exhibited uniformity in both the X and Y directions as illustrated in Figure 4. The second receiver was placed 0.5 cm below the sample to assess the beam properties after it passed through the sample. The output power measured by this receiver was 4.59 W, indicating that the sample absorbed some of the incoming laser light. Notice the droplet acts as a lens as the light passes through the sample. The beam diameter at the position of the receiver was 6.12 mm (measured at $1/e^{2}$ intensity) and the beam exhibited uniformity in the X and Y directions.

**FIGURE 5:**
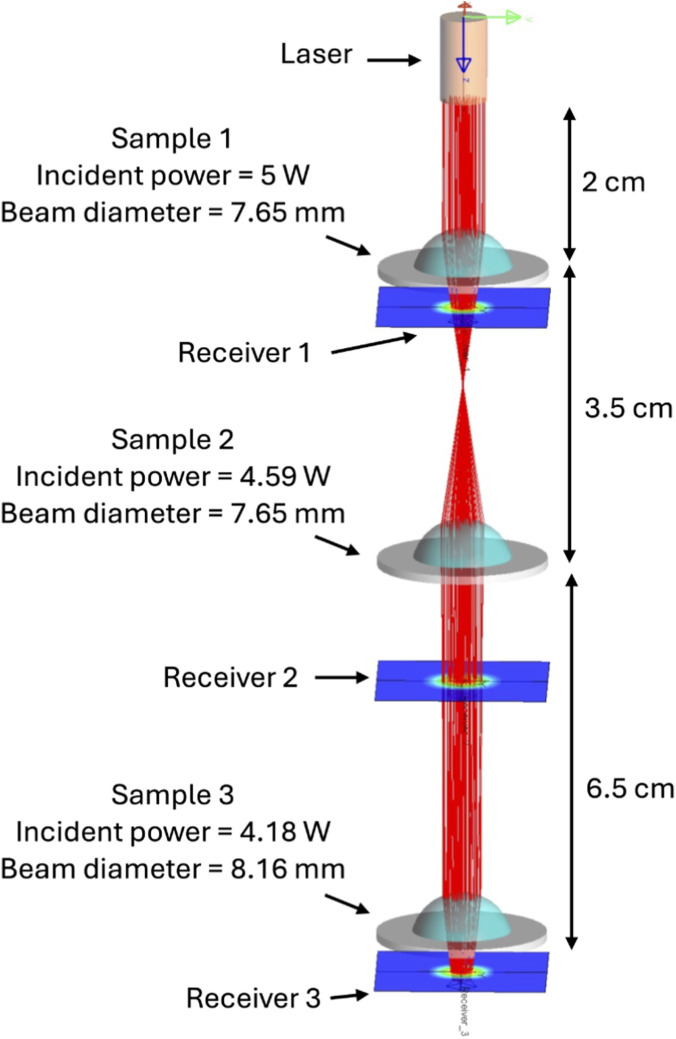
Geometry illustrating simultaneous processing of three samples in a vertical stack. The initial input power measured at the first receiver was 5 W. The blue rectangles are the receivers used in the simulation to calculate the laser power and beam profile at the different positions.

Less than 10% of the incident light is absorbed by the sample, suggesting that multiple samples can be processed simultaneously. To test this, light propagation through three samples was simulated in LightTools. Figure 5 shows the model of the laser passing through three droplets aligned in a vertical stack. The first sample is the same one modeled for the single sample setup discussed previously. The power incident on the first sample was 5 W and the beam diameter was 7.65 mm (measured at $1/e^{2}$ intensity). The second sample was placed 3.5 cm below Sample 1. Sample two was specifically placed at this position so that the beam diameters for Samples one and two were the same (7.65 mm, measured at $1/e^{2}$ intensity). The power incident on Sample two was 4.59 W. Notice that the beam is approximately collimated after passing through Sample 2. The third sample was placed 6.5 cm below Sample two and the power incident on this sample was 4.18 W. The beam size at Sample three was 8.16 mm (measured at $1/e^{2}$ intensity), which is slightly larger than the beam size used for the other two samples. An additional optic could be placed before Sample three to ensure that the beam size at Sample three exactly matched the spot size incident on Samples one and 2. The power measured after the light passed through Sample three was 3.83 W. The remaining power could be used to process additional samples in a vertical stack.

LightTools simulations were used to estimate the spatial distribution of the laser power transmitted through and deposited onto each samples in the stacked configuration. While this optical simulation provides valuable information about the relative power deposition from sample to sample in the stack, it does not account for the resulting thermal response in each sample. Because the laser energy absorbed by each sample in a stack differs, the associated temperature changes will also vary. Achieving uniform drying with minimal temperature differences between samples is crucial to ensure consistent sample quality and synchronized drying times. To address this, the lumped-parameter thermal model was used to quantify drying dynamics as a function of laser power.

### 3.2 How incident laser power affects the maximum temperature of the sample during LAD processing

To evaluate multiple sample processing designs, it was necessary to characterize how incident laser power influences both the maximum temperature achieved by each sample and the time required for complete water removal. In a stacked configuration, the first sample receives the greatest incident power, leading to a higher maximum temperature and shorter drying time, while the final sample receives the least power, resulting in a lower temperature and extended drying duration. An optimal incident power must therefore balance these competing needs: the first sample must not overheat and risk damage, while the last must reach a sufficient temperature to allow complete drying.

**FIGURE 6:**
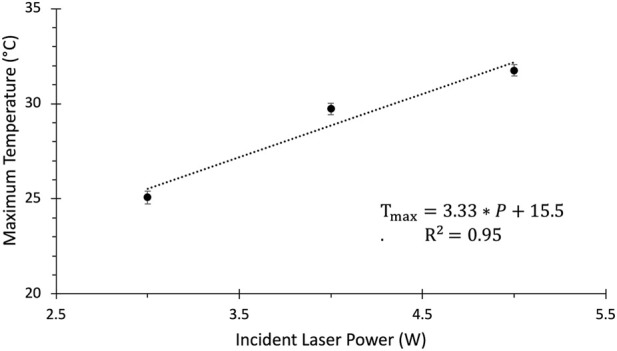
Maximum sample temperature ( ° C) *versus* incident laser power (W). The data points represent the mean maximum temperature ( N=3 ; ± SEM) for each power setting. A linear relationship was observed $\left(R^{2}=0.95\right)$ . The sample temperature scales predictably with laser power.

The maximum temperature reached during processing, $T_{\max}$ , was measured using the thermal camera across three incident laser powers to empirically derive a functional relationship between laser power and the maximum sample temperature. $T_{\text{max}}$ was measured at three different power settings: 3 W, 4 W, and 5 W (N = 3 for each power). The initial temperature of the sample for each power was the same ( $T_{i}=15.0{}^{\circ}\pm 0.1$ °C) to ensure easy comparison of the thermal curves. Figure 6 presents the graph of $T_{\text{max}}$ as a function of laser power. The relationship between laser power and $T_{\text{max}}$ was found to be approximately linear. A linear regression was performed, resulting in an empirical equation, which can be used to predict $T_{\text{max}}$ for any given incident laser power, P .

$$T_{\text{max}}\left(P\right)=3.33\cdot P+15.52\;\left(R^{2}=0.95\right)$$ (5)

As expected higher incident laser power results in a larger maximum temperature. This relationship allows estimation of sample temperatures for any incident laser power. It is especially useful for powers P>5W that cannot currently be tested in the experimental setup.

### 3.3 How incident laser power affects LAD processing time

**FIGURE 7:**
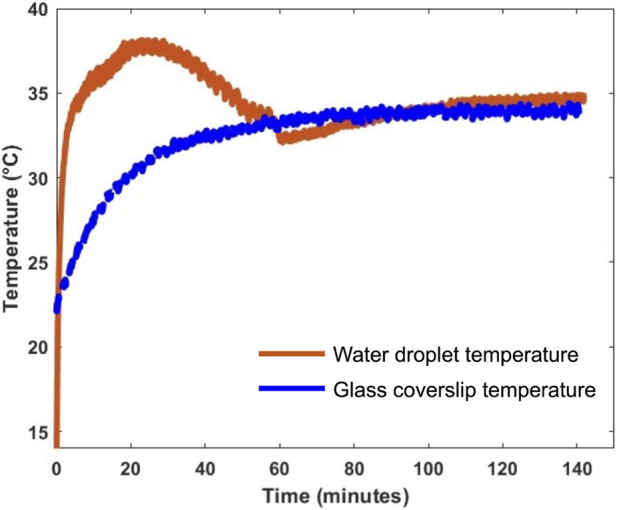
Representative experimental temperature *versus* processing time for a water droplet LAD processed on a coverslip (orange) and for a blank coverslip (blue).

The thermal model was used to predict the time required to remove all water from the sample (referred to as the processing time, $t_{p}$ ) using Equation 4. The first step in this analysis was to estimate the absorption efficiency of water, $a_{w}$ . To determine this parameter, the analysis focused on the initial heating phase immediately following the onset of laser irradiation. Figure 7 presents an experimental plot of $T_{\text{droplet}}$ and $T_{\text{coverslip}}$ *versus* time, obtained with the thermal camera during LAD processing. During the first minute of exposure, the rapid temperature increase observed in the droplet suggests that direct laser absorption is the dominant mechanism governing energy input and temperature rise. At this early stage, heat loss mechanisms, such as convection, evaporative cooling, and conduction to or from the substrate, are minimal and can be reasonably neglected. Consequently, the lumped energy balance equation for the droplet can be simplified by excluding all loss terms (see Equation 6).

$$\frac{dT_{w}}{dt}=\frac{a_{w}S_{w}}{\rho_{w}c_{pw}V_{w}}$$ (6)

From this expression, a first-order estimate of $a_{w}$ was obtained by equating the absorbed laser power to the rate of internal energy increase over a short time interval, $t_{1}$ (the first 1 minute of heating; see Equation 7).

$$a_{w}S_{w}\approx \rho_{w}c_{p,w}V_{w,0}\cdot \frac{T_{w}\left(t_{1}\right)-T_{w}\left(0\right)}{t_{1}}$$ (7)

where $V_{w,0}$ is the initial volume of water, $T_{w}\left(t_{1}\right)$ is the water temperature at 1 min, $T_{w}\left(0\right)$ is the initial temperature of the water, and $t_{1}$ is 60 s. To calculate the absorption efficiency, the measured initial temperature of the droplet, the temperature recorded at 60 s for the droplet, and the known laser power input were used. The value of $a_{w}$ was estimated from 3 W, 4 W and 5 W incident power thermal curves. These were combined with known physical parameters such as material density, specific heat capacity, and volume (see Table 2) to calculate the absorption efficiency. The volume of the droplet was assumed to be constant across this small time increment. The mean calculated value of $a_{w}$ and the SEM (N = 11) is listed in Table 2. This value for $a_{w}$ was then used in subsequent analysis.

**TABLE 2:** Values of constants used in the model.

Constant	Value
$\rho_{w}$	998.2 kg/m^3^
$c_{pw}$	4182 J/(kg ⋅ K)
$a_{w}$	0.033 ± 0.003
$h_{fg}$	2.454 x $10^{6}$ J/K

The next step was to find an expression that described the change in volume of the droplet with time. By rearranging Equation 4 and applying the chain rule, an expression for $\frac{dV}{dt}$ , the rate at which the volume of the droplet changes during processing, was derived (see Equation 8).

$$\frac{dV_{w}}{dt}=\left(\frac{a_{w}S_{w}}{\rho_{w}c_{pw}}-V\frac{dT}{dt}\right)\left(\frac{c_{pw}}{Tc_{pw}-h_{fg}}\right)$$ (8)

**FIGURE 8:**
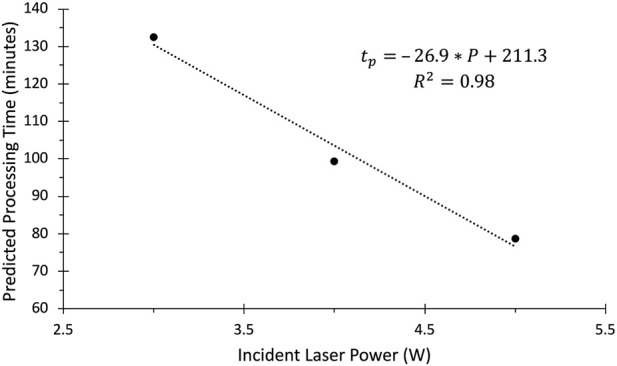
LAD processing time as a function of laser power predicted by the lump-parameter thermal model using dT/dt measured with the thermal camera as input.

Temperature *versus* time data (dT/dt) measured by the thermal camera was used as input to this equation. The latent heat of vaporization of water was assumed to be $h_{fg}=2454\;kJ\;kg^{-1}$ , as reported by Chen *et al.* for water at 20 °C and atmospheric pressure (Chen et al., 2006). The model was solved numerically in MATLAB using the ode45 solver. We simulated $\frac{dV}{dt}$ for three different laser powers (3 W, 4 W, and 5 W), using the corresponding experimental temperature data for each case. From these simulations, we determined the time at which the predicted droplet volume reached zero. Figure 8 displays a plot of $t_{p}$ as a function of laser power. The relationship is approximately linear. A linear regression yielded an empirical formula that can be used to estimate $t_{p}$ in minutes for any incident laser power

$$t_{p}=-26.95\cdot P+211.34\;\left(R^{2}=0.98\right)$$ (9)

where P is the incident laser power in Watts. As expected, the processing time increases for lower laser powers.

### 3.4 Model predictions for stacked sample processing

The predictive equations (Equations 5, 9) were used to estimate the maximum number of samples that can be simultaneously processed while satisfying constraints on realistic sample temperature and total processing time. Each sample absorbs approximately 3% of the incident laser power (based on our estimate of $a_{w}$ ). Although the absorption efficiency is relatively low, significant optical losses occur as the laser propagates through the stacked samples. At each interface, approximately 4% of the incident power is lost due to Fresnel reflection. A cumulative reduction of about 16% per sample was assumed. An upper limit on the incident laser power for the first sample in the stack was determined by setting the maximum allowable temperature, $T_{\text{max}}$ , to 37 °C. This threshold is near physiological temperature and is appropriate for preserving thermally sensitive biomolecules. According to Equation 5, a laser power of 6.4 W ensures that $T_{\text{max}}$ remains within this safe range. A lower limit for the required laser power for the last sample in the stack was then established by setting the maximum allowable processing time to 3 h (180 min). Based on Equation 9, the final sample in the stack must receive a minimum of 1.2 W to achieve complete drying within this time constraint. Considering the 16% power reduction per sample, the total number of samples that can be processed in a single stack before the incident power drops below 1.2 W was calculated. This analysis predicts that up to 10 samples can be dried simultaneously without exceeding the thermal or temporal limits. This predictive analysis provides a practical framework for scaling up sample processing while minimizing the risk of thermal damage.

### 3.5 Evaluation of protein stability following multi-sample LAD

To evaluate the feasibility of multi-sample LAD processing, we utilized the LightTools and lumped-parameter thermal models to predict maximum sample temperatures and processing durations for the three-sample system shown in Figure 5. The model predicted that using an incident power of 5 W (currently available in the experimental setup) for Sample one would ensure that the sample temperature remained below 37 °C and the required processing time would be 77 min (Table 3). The last sample in the stack (Sample 3) would also remain below 37 °C and have a processing time of approximately 100 min.

**TABLE 3:** Predicted maximum sample temperatures and processing times for the prototype multi-sample system shown in Figure 5.

Sample number	Input power	Predicted $T_{\max}$	Predicted processing time
1	5.0 W	32.2 °C	77 min
2	4.59 W	30.8 °C	88 min
3	4.18 W	29.5 °C	99 min

**FIGURE 9:**
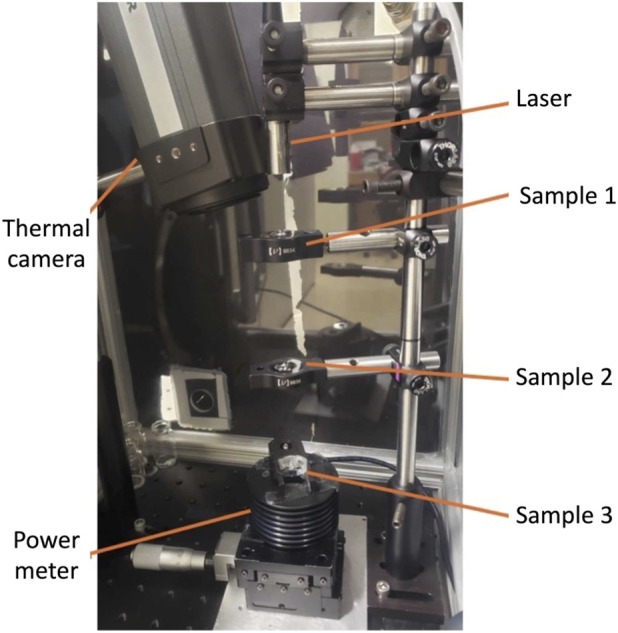
Proto-type for simultaneous multi-sample processing.

A prototype capable of simultaneous multi-sample processing was constructed to validate these simulations. A droplet-on-coverslip geometry was selected to ensure direct parity with the optical and thermal model parameters. Using the configuration illustrated in Figure 9, three samples containing IgG as a test biologic were processed in parallel. Although the model predicted drying at 100 min, the experimental run was extended to 140 min to fully characterize the thermal plateau and confirm the transition into the region of constant temperature (Figure 7). As established in previous work, reaching this plateau signifies the cessation of significant evaporation (Anh Lam et al., 2022; Furr et al., 2024; Tsegaye et al., 2024). The temperature of Sample one remained below 37 °C, and the thermal curve reached its plateau by the 100-min mark (80 ± 5 min). Thermal histories for all samples in the stack were similar to those seen in Figure 7. Samples two and three reached the temperature plateau slightly later than Sample 1; Sample two reached the plateau at 90 ± 5 min and Sample three at 100 ± 5 min. These processing times are consistent with the predictions of the model (see Table 3).

PLI was employed to confirm that the matrix remained amorphous immediately after processing and after 2 months of storage at room temperature (20 °C). Recall that crystallization signals matrix instability. For the trehalose matrix, stability in the amorphous state requires drying to approximately 2% water content by mass. A lack of crystallization indicates that these moisture contents were successfully achieved. Analysis *via* PLI indicated that the vast majority of samples (14 out of 15) showed no evidence of crystallization following both processing and storage. This suggests that the amorphous matrix remained stable and provided robust protection for the embedded protein. A single sample did crystallize during the storage period. This specimen was subsequently used to evaluate the potential damage to the embedded biologic in this destabilized matrix. The crystallization of this outlier was likely triggered by a sample impurity acting as a nucleation seed, an interaction with the cover glass (a phenomenon observed in previous work), or localized insufficient dehydration in that specific sample.

**FIGURE 10:**
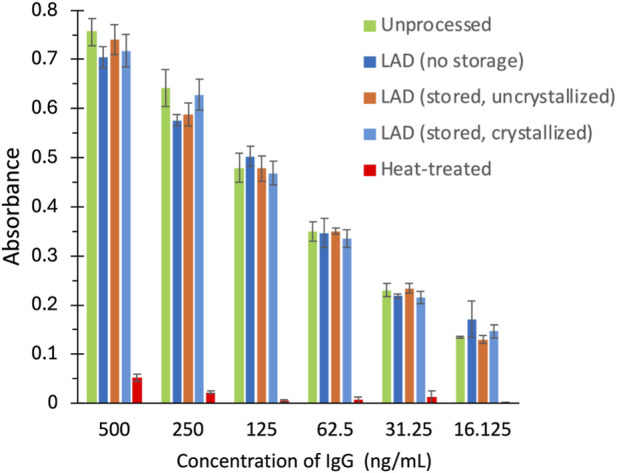
ELISA assay results for IgG samples. Absorbance values were similar among the unprocessed and all LAD-processed groups. However, the heat-treated sample showed significantly lower absorbance, indicating irreversible protein degradation.

ELISA assays were performed to evaluate the *in vitro* function of IgG. Five different sample types were tested: unprocessed IgG (Unprocessed, N=6 ); LAD-processed samples tested immediately after drying (LAD no storage, N=4 ); LAD-processed samples that remained uncrystallized after 2 months in storage (LAD stored, uncrystallized, N=6 ); LAD-processed samples that crystallized after 2 months in storage (LAD stored, crystallized, N=6 ); and a heat-treated sample (Heat-treated, N=8 ) used as a negative control. Figure 10 shows the ELISA results. The absorbance values across all LAD-processed samples were similar. In contrast, the heat-treated IgG exhibited significantly lower absorbance compared to all other groups, suggesting irreversible degradation.

To quantify these findings, a statistical analysis was performed. The Lilliefors test indicated that the data for all concentrations except the lowest (16.125 ng/mL) were normally distributed. Consequently, this lowest concentration was omitted from further statistical analysis. A Levene’s test revealed differences in variance across the groups. Therefore, a Welch’s ANOVA was utilized, with *post hoc* comparisons performed using the Games–Howell test. No statistically significant difference was observed between the unprocessed IgG and any of the LAD-processed groups (p>0.05) . These results indicate that the drying process has minimal impact on the functional binding of IgG. The heat-treated samples exhibited significantly lower absorbance (p<0.05) than all LAD-processed groups, confirming significant damage to the protein under those conditions.

**FIGURE 11:**
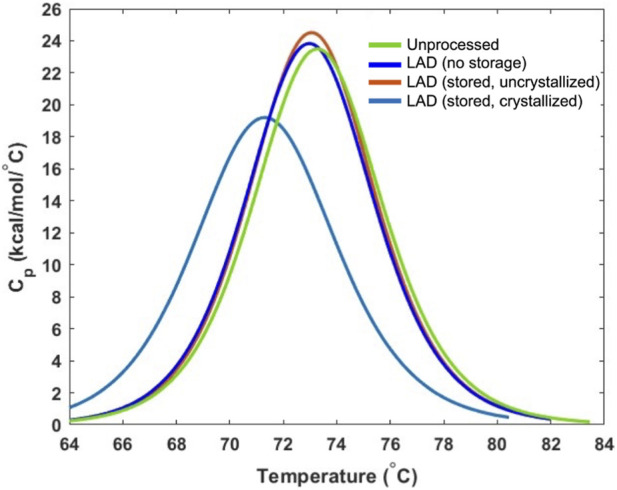
DSC curves of unprocessed, LAD-processed (no storage), LAD-processed (stored, no crystallization) and LAD-processed (stored, with crystallization) samples.

To evaluate the structural stability of IgG following multi-sample LAD processing, DSC was performed on four groups: unprocessed samples (Unprocessed; N = 3), LAD-treated samples analyzed immediately after processing (LAD no storage, N = 3), non-crystallized LAD-treated samples stored for 2 months at 20 °C (LAD stored uncrystallized, N = 3) and the crystallized LAD processed sample (LAD stored crystallized, N = 1). The calorimetric curves are shown in Figure 11 and thermodynamic quantities derived from these curves are listed in Table 4. LAD-processed samples analyzed immediately after drying showed close agreement with the stock protein, exhibiting sharp unfolding transitions centered near 73 °C. This indicates that multi-sample LAD processing preserves the native thermal behavior of the protein. Similarly, non-crystallized samples that had been stored for 2 months retained $C_{p}$ profiles and unfolding enthalpies (ΔH) similar to the stock, demonstrating that structural integrity was maintained during storage when crystallization was avoided. In contrast, the stored sample that underwent crystallization showed deviations, including a broader peak, earlier onset temperature, and a 10.09% reduction in ΔH , consistent with partial protein denaturation. These findings confirm that multi-sample LAD processing is compatible with maintaining biologic stability, provided crystallization is avoided during and after processing. The DSC analysis of the crystallized LAD samples suggested a shift in global structural properties, likely due to conformational stress induced by the crystal lattice. However, the ELISA results demonstrate that the IgG retained its specific binding integrity. This suggests that the lattice-induced constraints detected by thermal analysis did not translate to a loss of paratope functionality in this case.

**TABLE 4:** Thermodynamic parameters for IgG as determined with DSC.

Sample type	ΔH (kcal/mol)	$T_{m}$ (°C)	% change $T_{m}$	% change ΔH
Unprocessed	$1.50\times 10^{5}$	73.3 ± 0.1	-	-
After LAD	$1.51\times 10^{5}$	72.6 ± 0.1	1.0	0.9
2 months storage (No crystallization)	$1.53\times 10^{5}$	73.1 ± 0.1	0.3	2.1
2 months storage (Crystallization)	$1.35\times 10^{5}$	71.3 ± 0.1	2.7	10.9

## 4 Discussion and conclusion

The LightTools simulations confirmed that a multi-sample vertical stack is feasible for the LAD process. By modeling the laser path through a three-sample configuration, we determined sufficient power penetrates the stack to drive evaporation in successive samples. Furthermore, the model provides a way to predict the incident power at each sample in the stack, which is critical for predicting the maximum temperature and drying kinetics. This finding provides the foundation for scaling the system, suggesting that a single energy source can effectively process multiple samples.

The lumped-parameter thermal model provided a predictive framework for understanding the thermal history and drying kinetics of each sample in the stack. An empirical relationship between incident power and maximum sample temperature was established, allowing for the identification of power thresholds required to keep samples below specific thermal degradation limits. In addition, we could predict the drying time for a sample as a function of incident laser power. The model predicts that an incident power of 6.2 W is sufficient to process a 10-sample vertical stack while maintaining a safe thermal profile and completing processing in under 3 hours.

Using LightTools, we designed a system to process three samples in a vertical stack with 5 W incident on the first sample. We combined the output of these optical models (incident power per sample) with the lumped-parameter thermal model to predict the thermal histories and drying of these three samples. A proto-type was then constructed and tested. The theoretical predictions for temperature and processing time were in good agreement with experimental data. This correlation validates the thermal model as a reliable design tool for optimizing throughput while ensuring the safety of thermally sensitive biologics.

The performance of the multi-sample system was experimentally verified using IgG as a model therapeutic protein. The cumulative laser exposure required for batch processing does not induce structural or functional damage. ELISA and DSC analyses showed that the rehydrated protein maintained its specific binding and structural integrity, both immediately following LAD processing and after 2 months of storage at 20 °C. These results confirm that the serial irradiation utilized in the vertical stack configuration creates a stable, high-quality amorphous glass comparable to single-sample LAD. Furthermore, the lack of degradation over the storage period validates the efficacy of the multi-sample laser method for long-term ambient preservation of biologics. While this study utilizes Human IgG as a model protein, the established optical-thermal framework is broadly applicable to other biologics, as the laser-matter interaction is dominated by the absorption characteristics of the trehalose-water matrix.

By demonstrating that a single laser source can effectively stabilize a vertical stack of samples, we have identified a clear pathway for scaling the LAD process. This multi-sample configuration increases throughput without requiring complex optical arrays or excessive energy consumption. Furthermore, the predictive accuracy of our combined models allows for the rapid optimization of scale-up parameters. Ultimately, this approach moves LAD closer to a high-throughput industrial reality, offering a viable alternative to processes like lyophilization.

Future work will focus on further increasing processing throughput. We are currently adapting the optical and thermal models to accommodate pharmaceutical glass vials. Additionally, we are exploring configurations that utilize multiple vertical stacks to enable high-capacity batch processing. The scalability of this vertical LAD platform is governed by cumulative laser attenuation and Fresnel reflections at each air-material interface, which can eventually limit drying uniformity as stack height increases. Transitioning this proof-of-concept to industrial fill-finish production will involve collaboration with industry partners to address engineering requirements, such as high-precision optical alignment and the integration of uniform convective cooling across all stacked layers. These advancements will refine the system’s efficiency and validate its utility as a scalable, high-throughput alternative for the preservation of biologics.

## Data Availability

The raw data supporting the conclusions of this article will be made available by the authors, without undue reservation.
